# A Metal‐Free Electrode: From Biomass‐Derived Carbon to Hydrogen

**DOI:** 10.1002/cssc.202000714

**Published:** 2020-06-10

**Authors:** Yuxiao Ding, Mark Greiner, Robert Schlögl, Saskia Heumann

**Affiliations:** ^1^ Max Planck Institute for Chemical Energy Conversion Stiftstrasse 34–36 45470 Mülheim an der Ruhr Germany; ^2^ Fritz Haber Institute of the Max Planck Society Faradayweg 4–6 14195 Berlin Germany

**Keywords:** biomass, carbon, hydrogen production, renewable energy, water splitting

## Abstract

Hydrogen is the emission‐free fuel of the future if produced from non‐fossil sources. Biomass gasification or electrolysis of water are possible clean routes. For a global application, the material solution for the electrodes must be sustainable, scalable, and relatively inexpensive compared to the current precious metal‐based electrodes. A key requirement to sustainable and green energy systems is that all harmful or rare resources utilized in the process must be part of a closed material cycle. Here, a carbon‐based electrode for hydrogen production is presented that can be part of a closed material cycle if produced from biomass. Continuous hydrogen production takes place at the cathode through catalytic water splitting during the oxygen evolution reaction (OER), while intentionally allowing the decomposition of the electrode into CO_2_ analogous to the process of natural biomass decomposition. This strategy of a sacrificial electrode could provide a scalable and low‐cost material solution for hydrogen production from renewable energy sources. The theoretical and technical feasibility of using carbon to produce hydrogen is demonstrated, and it is shown that chemical modification can further improve the performance characteristics towards the catalytic process. Combined with renewable energy derived electricity, this idea offers a real option for future energy systems.

## Graphical abstract

**Make a sacrifice**: Hydrogen production by electrolysis requires sustainable, scalable, and relatively inexpensive electrodes. Carbon‐based electrodes obtained from waste biomass meet these requirements. Not only the theoretical but also the chemical and technical feasibilities are discussed and experimentally demonstrated. The use of sacrificial carbon electrodes from biomass, operated with renewable energies, reveals a real solution for future energy systems.

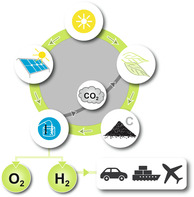

## Introduction

The energy supply of the future is based on renewable sources. Although solar radiation and wind are endlessly available from nature, their energy is not directly usable in most cases. These primary sources are generally converted into other forms, and then either transported directly to the end user, stored, or converted to another energy vector more suited to the needs of the end user.^1^, ^2^ Chemical energy is among the most useful of vectors. Not only does it represent the main energy feedstock of all forms of synthesized material products, it also represents a very effective long‐term storage medium because a wisely chosen molecule will not naturally decompose at an appreciable rate.^3^ One of the most common approaches to storing renewable energy in chemical form is using electricity to split water into H_2_ and O_2_.^4^, ^5^ In this case, the energy vector is H_2_, and it is desirable because it contains a high energy density per weight and is a vital feedstock for chemical industry.^6^ The main challenge of large‐scale water electrolysis is the material solutions of the electrodes.^7^, ^8^, ^9^, ^10^, ^11^ Unfortunately, the best materials for activity and stability are based on iridium or platinum, which are among the rarest and most expensive elements on the planet.^12^ The vast majority of known electrode materials are ineffective at water splitting or dissolve when exposed to the required electrochemical conditions.^13^ Organic molecules involved in the electrolyte decrease the energy consumption,^14^, ^15^ but the separation of water phase and organic phase restricts the scalability. Furthermore, consumed organic molecules have to be continuously refilled owing to their irretrievable consumption. To fulfill the requirement of sustainability, it must necessarily involve only closed material cycles. This means that all products of the process must eventually be re‐transformed into starting materials. This “closed material cycle” requirement is particularly pressing when the materials in question are either rare starting materials or toxic byproducts. These factors dictate the profitability and eco‐friendliness of the energy system.

**Figure 1:**
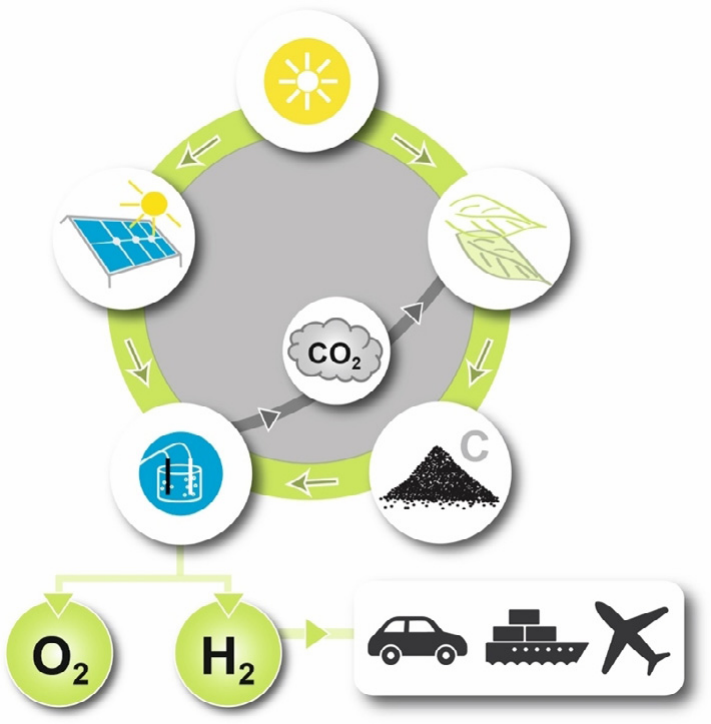
Closed carbon cycle when biomass is used as renewable precursor for sacrificial carbon‐based electrodes. The sun provides energy to convert CO_2_ into biomass as well as electricity for the water‐splitting process to produce “green” hydrogen.

We present a metal‐free carbon electrode made from abundant biomass materials that exhibits high electrochemical efficiency, while, owing to the choice of material, the problem of corrosion is addressed by intentionally allowing it to occur. The hydrogen production on the cathode side takes place unhindered, no matter what kind of reaction takes place at the anode—either the catalytic oxygen evolution reaction (OER, water oxidation) or a mild combustion of carbon (carbon oxidation). This “acceptable corrosion” strategy is possible because the electrodes are carbon‐based and synthesized from waste biomass. In contrast to the commercially used electrodes (IrO_*x*_, RuO_*x*_) or transition metal‐based materials,^7^, ^9^, ^16^, ^17^, ^18^, ^19^, ^20^, ^21^ the pure carbon‐based electrode can be used as a sacrificial electrode owing to the sustainability of its supply and its scalability. Figure 1 illustrates the proposed energy and materials cycle. Solar energy is used to grow biomass, which is subsequently processed into biofuels. The processing of biomass leaves behind between 10–50 wt % unusable organic matter as a byproduct, generally in the form of humins and char.^22^ The unused biomass is converted into carbon electrodes and then utilized in electrolyzers, in which electricity generated by renewable sources drives water electrolysis. The carbon‐based electrodes eventually corrode through oxidation into CO_2_, which is generated as a byproduct at a rate no greater than it is consumed in the growing of biomass. Note that this condition is met as long as only biomass is used to make the carbon electrodes. In essence, an OER electrode simply provides an avenue through which electrical energy can be transferred into chemical bonds. Whereas most materials cannot withstand the conditions required for this process to happen, by fabricating sacrificial electrodes out of materials that would otherwise be burned we put waste materials to a more prudent use. In this case, both energy from electricity and energy from carbon is stored in the form of hydrogen.

## Results and Discussion

Although much previous work has focused on understanding the degradation mechanism of carbon materials under electrochemical conditions,^23^ it appears that corrosion is an inevitable process, and it has been labeled as the major drawback of using carbon electrodes over noble metal electrodes. However, there are some interesting attributes of carbon electrodes that could make them more appealing if properly exploited. For instance, the oxidation potential of carbon is 0.207 V_RHE_ (RHE: reversible hydrogen electrode),^24^ which means that weakly bonded carbon atoms within solid carbon structures could be oxidized at a potential lower than the theoretical 1.23 V_RHE_ for the OER process. This could provide a lower‐energy pathway to produce hydrogen, meaning that lower potentials are needed to drive the reaction. Although the standard oxidation potential of carbon materials is not fixed and is highly dependent on the carbon structure, it is generally much lower than the OER process (Figure S1 in the Supporting Information). The carbon oxidation process is a lower‐energy process than the oxygen evolution process, suggesting carbon oxidation is more favorable than water oxidation when electrolyzing water to produce hydrogen. Nevertheless, the catalytic OER is the desired process because it does not lead to degradation of the electrode and one‐time stochiometric hydrogen production. It is also worth mentioning that there are reported cases of metal‐free carbons as highly efficient anodes for water splitting. The high activity does not come from carbon itself because it experiences self‐oxidation before OER happen. The oxygen functional groups on the highly graphitic carbon surface have the ability to trap ppb levels of iron impurity from the electrolyte.^25^ The newly formed iron species on the carbon surface provide the real active sites for OER and protect the carbon from deep oxidation.^25^ The carbon oxidation and impurity issues should also be considered in those heteroatom‐doped carbon materials.

**Figure 2:**
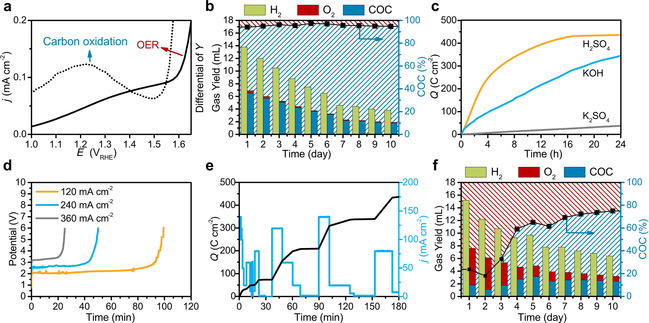
a) Current–potential curve of a carbon as electrode and the relative differential curve (dashed line). b) Daily gas collection in a two‐electrode cell: hydrogen at the cathode; oxygen and the carbon oxidation contribution (COC) at the anode. Red‐shaded areas demonstrate the hydrogen production from water oxidation (catalytic process), whereas blue‐shaded areas are the hydrogen production from carbon oxidation. c) Charge collection of the carbon pellet in acidic (H_2_SO_4_, pH 1), alkaline (KOH, pH 13), and neutral (K_2_SO_4_, pH 7) electrolytes. d) Stability of the carbon pellets in 1 m KOH at current densities of 120, 240, and 360 mA cm^−2^. e) Performance of the carbon pellet under dynamic potential variations to mimic the flexibility of solar‐derived electricity. f) Daily gas collection in a two‐electrode cell of the nitrogen‐containing carbon pellet.

**Figure 3:**
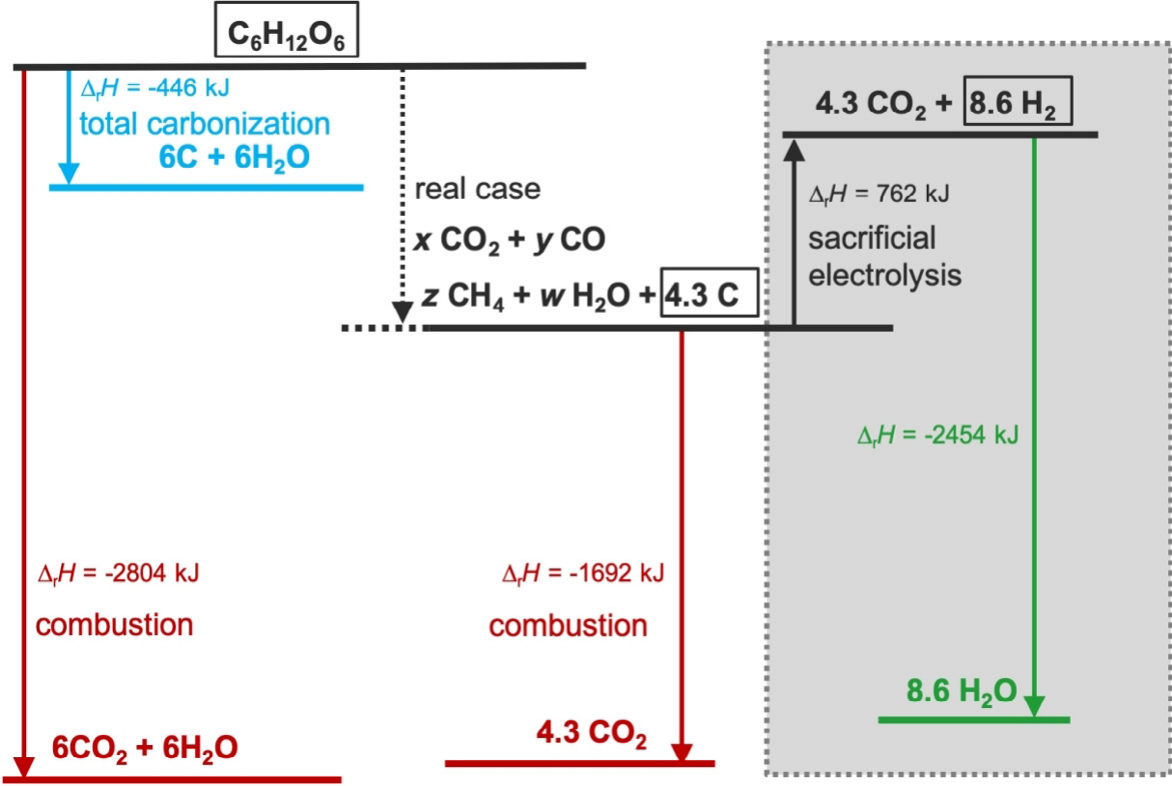
Energy diagram of glucose conversion. Red=combustion, blue=carbonization, black dotted=hydrothermal process, black=sacrificial electrolysis (hydrogen production+carbon corrosion), green=hydrogen combustion, grey=one‐off process in case of 100 % sacrificial electrolysis.

A measured oxidation curve of a carbon sample is depicted in Figure 2 a to illustrate basic electrochemical properties of carbon (see Methods in the Supporting Information). The carbon material is a representative of a non‐graphitic powder (annealed hydrothermal carbon) and was dropcoated onto a glassy carbon electrode in a three‐electrode system in Ar‐saturated 0.1 m KOH (see Methods in the Supporting Information). The two peaks of the differentiated curve (dashed line) confirm the catalytic oxygen evolution process (onset potential 1.52 V_RHE_) and the carbon oxidation process (onset potential 1.02 V_RHE_). In contrast to the OER process, in this reaction the carbon‐based anode is consumed, and stoichiometric production of hydrogen takes place at the cathode. A major concern with using sacrificial carbon electrodes might be that producing H_2_ is less economical than the direct use of carbon as solid fuel in combustion engines or for biomass gasification. Established processes convert the biomass directly in volatile products such as gases, fuels or carbon materials by stoichiometric conversion only. The procedure is therefore limited by the one‐off consumption of biomass. If one molecule of glucose, which is used as representative for biomass, is totally oxidized, the heat of combustion is around 2800 kJ mol^−1^ (Figure 3). In contrast, a total carbonization would generate around 450 kJ mol^−1^ (blue), whereas during a typical hydrothermal treatment (see Methods in the Supporting Information) of glucose, which is applied to obtain the carbon‐based electrodes, around 4.3 mol of carbon and additional volatile gases are generated. The released energy consequently lies between the first two processes (black dotted). An application of the annealed hydrothermal carbon as sacrificial electrode and the worst possible assumption that no electrocatalytic OER but 100 % mild oxidation takes place (grey area) would not lead to a worse energy balance than the direct combustion of the glucose without even considering the Carnot efficiency. An increase of the ratio towards electrocatalytic hydrogen production process away from mild oxidation would result in a much better energy balance because hydrogen production could occur several times per carbon atom. In addition, the combustion of carbon is limited by the Carnot efficiency, in which only 35–40 % of combustion energy can be efficiently used. In contrast, in the electrochemical oxidation process, theoretically 100 % of the energy is available for the electrochemical hydrogen production process.

Plant biomass has been used already to produce low‐cost carbonaceous materials, demonstrating the opportunity for large‐scale production.^26^, ^27^ To meet a scalable, independent, and long‐term application, a technique to press the powdery carbon into pellets without binders was developed (see Methods in the Supporting Information). The electrochemical tests with the carbon pellets were performed in a two‐electrode cell (Figure S2 in the Supporting Information), and the gaseous products were collected. Figure 2 b shows the gas accumulation over a 10‐day period. Detailed characterization of the material is shown in Figure S3 in the Supporting Information. The cathode side continuously produced hydrogen, whereas the anode side produced a marginal amount of oxygen. The main product at the anode site was CO_2_, which was directly dissolved in the electrolyte in the form of carbonate. Carbonate can be directly detected by MS (Figure S4 in the Supporting Information). In general, recycling of the pure CO_2_ from the electrolyte is much easier than controlling the greenhouse gas from fossil combustion from the atmosphere, which is mixed with quite a few other gases. The contribution of the catalytic production was stable at around 5 % in comparison to the carbon oxidation contribution (COC), which settled at 95 % (Figure 2 b). The total gas production decreased owing to the consumption and detachment of small carbon fragments from the pellet (Figure S5 in the Supporting Information). The lifetime of an electrode at industrial scales should cover several thousand hours and would require a continuous feed of the sacrificial electrode.

Performance modifications of the carbon pellets can be induced by simply changing the electrolyte. Charge collections at carbon anodes in alkaline, neutral, and acidic media reveal different electrochemical behavior (Figure 2 c). In alkaline media, the carbon electrode is continuously oxidized to form carbonate and produces a constant current to generate hydrogen at the cathode side. In neutral media, the reaction is very slow, whereas in acidic media, the carbon electrode is functionalized to form oxygen functional groups. The functional groups cannot be further oxidized and protect the carbon surface (Figures S6–S8 in the Supporting Information). To recover the activity of the electrode, the electrochemically generated functional groups can be removed by annealing or changing the potential.

Electric current densities of 120, 240, and 360 mA cm^−2^ were applied to mimic industrially relevant conditions (Figure 2 d). The carbon electrodes showed constant potentials at 2, 2.5, and 3.1 V_RHE_ until the pellets fell apart, which was caused by the large amount of gas formation. Making larger domains of carbon pieces can overcome the problem (Figures S9 and S10 in the Supporting Information). Increasing the graphitic proportion in the biomass‐derived carbon structures can be achieved by increasing the synthesis temperatures or adding catalysts during the synthesis. The behavior of carbon electrodes under dynamic potential changes was investigated to simulate the fluctuating electricity supply of solar panels (Figure 2 e, Figure S11 in the Supporting Information). The pellet showed charge‐transfer rates in accordance with the applied currents, demonstrating that carbon‐based electrodes can withstand the dynamic fluctuations. With the current density of 120 mA cm^−2^ at 2 V_RHE_, the exposed area of the carbon should be around 416 m^2^ to set up a 1 MW electrolyzer. With the current 0.77 mm thickness and 0.78 g cm^−3^ density of the pellet, the amount of the carbon would be around 249 kg to fulfill this. To construct an electrolyzer of appropriate dimensions, one would have to increase the surface area, which can be achieved, for instance, with activation techniques or templating.

Apart from the technical feasibility of the carbon electrodes, the flexibility for chemical modification presents many possibilities to reduce the rate of carbon consumption. A simple and scalable modification of the carbon material by incorporation of nitrogen was performed to examine the effect of functionalizing carbon‐based electrodes (see Methods in the Supporting Information). The resulting nitrogen‐doped carbon performed remarkably better than the pure carbon counterparts (Figure 2 f). Within the first three days the catalytic contribution reached values around 80 % in comparison to the mild oxidation (COC content). This results in a significantly higher stability of the electrode with consistent hydrogen production. The resistivities of the nitrogen‐containing carbon and the nitrogen‐free carbon are 50.5×10^−5^ and 45.4×10^−5^ Ω m, respectively. This indicates that the dopants have no big influence on the conductivity of the carbon. The hydrophilicities of the two samples are also similar. The water contact angle for the nitrogen‐containing sample is 56°, and that for the undoped sample is 48°. The surface functionality is quite different and causes relative work functions of 293.5 and 434.5 meV for the nitrogen‐containing and nitrogen‐free samples, respectively. Therefore, the change of the electronic structure caused by the nitrogen doping might be the main factor for the enhanced catalytic OER by a different mechanism on the surface. The effects of doping by heteroatoms or different metals on mass transport and catalysis will be studied in our following research.

In summary, a chemical energy‐conversion concept of using sacrificial carbon electrodes for the conversion of solar‐derived electricity to hydrogen by water splitting is proposed. The anodic biomass‐derived carbon electrode can fulfill either the classical catalytic water oxidation or a self‐oxidation. The technical feasibility is demonstrated in different electrolytes and under industrially relevant conditions of high, dynamic currents. The prospect of chemical modification shows big potential of improving electrode longevity. Thus, instead of using carbon as a direct energy carrier, the application of carbon as sacrificial electrode provides an industrial clean, scalable, and sustainable idea to obtain “green” hydrogen.

## Author contributions

Y.D. conceived the research, carried out material synthesis and basic characterization, and performed the electrocatalytic test. R.S. and S.H. supervised the study. The manuscript was written by Y.D. and S.H. and linguistically revised by M.G. All authors contributed to data analysis and manuscript revision.

## Conflict of interest

*The authors declare no conflict of interest*.

## Supporting information

As a service to our authors and readers, this journal provides supporting information supplied by the authors. Such materials are peer reviewed and may be re‐organized for online delivery, but are not copy‐edited or typeset. Technical support issues arising from supporting information (other than missing files) should be addressed to the authors.

Supplementary
