# Supplementary material for: A Metal‐Free Electrode: From Biomass‐Derived Carbon to Hydrogen
Source: ChemSusChem. 2020 Jun 10;13(16):4064–8. doi: 10.1002/cssc.202000714 (PMC7496841; doi:10.1002/cssc.202000714)
Supplement: Supplementary file 1 — Supplementary [file CSSC-13-4064-s001.pdf]

ChemSusChem

Supporting Information

A Metal-Free Electrode: From Biomass-Derived Carbon to Hydrogen

Yuxiao Ding,*[a] Mark Greiner,[a] Robert Schlögl,[a, b] and Saskia Heumann*[a]

Methods

1.1 Materials synthesis

1.1.1 Synthesis of the glucose derived carbon

The synthesis of the carbon materials was performed in 50 mL Teflon-lined autoclaves heated in a heating block system to 250 °C for 6 h. For a typical experiment, 30 mL of a 20 wt % glucose solution were prepared in aqueous solution.[1] The solution has a pH value of 6. After the hydrothermal treatment, the autoclaves were taken out of the heating device and cooled down over night. The solid product was filtered and washed with water thoroughly. The solid product was dried overnight in vacuum at 60 °C. The final product was named hydrothermal carbon precursor.

The obtained hydrothermal carbon precursor was removed to small quartz boats which were placed in the center of a larger quartz tube running through the center of a furnace. The furnace tube was vacuumed for 3 times to remove the inside air before the annealing process. Temperature programmed annealing processes were carried out according to the following procedures: annealing the hybrid from room temperature to 60 °C (Argon, 20 min), maintaining for 2 h; then heating to 1000 °C with a heating rate of 10 K/min, maintaining for 5 h. The oven was cooled down to room temperature without active cooling. The final products were obtained.

1.1.2 Synthesis of the carbon pellet

100 mg of the hydrothermal carbon precursor powder is pressed for 2 minutes under vacuum with a pressure of 10 tons. A pellet of 13mm diameter is obtained and then annealed in the furnace with the same procedure we used for the powder samples. The annealed pellets have weights of around 52.9 mg. The height of the pellets are around 0.77 mm with a diameter around 10.5 mm; the density is 0.78 g/cm^3.

1.1.3 Synthesis of nitrogen doped carbon pellet

Both starting materials 7.71 g glucose and 1 g urotropine (nitrogen source), are dissolved in 30 mL aqueous medium, filled in 50 mL Teflon lined autoclaves and treated hydrothermally at temperatures up to 250 °C for 6 h. The solid powder is filtered and washed with 10 L deionized water. The solid product was dried overnight in vacuum at 60 °C. Pellets were obtained in the same way as described above. The remaining mass of the final products is 50.2% of the precursor. The obtained pellet has a diameter of 10.06 mm, a height of 0.64 mm and a density of 1.01 g/cm^3.

1.2 Electrochemistry methods

Two different cells, one with three electrodes for typical measurement and one with two electrodes for industry technical feasibility test, are used for the measurement of the carbon materials.

1.2.1 Three-electrode cell

The three-electrodes system was controlled by using a potentiostat/galvanostat (BioLogic VSP, France). A platinized Pt wire as a counter electrode and a reversible hydrogen electrode (RHE, HydroFlex, Gaskatel GmbH) as a reference electrode were used. Electrochemical measurements comprise conditioning of work electrode, measurement of the open circuit potential (OCP), impedance spectroscopy (determination of iR-drop), cyclic voltammetry (CV, “activity”) and constant-potential chronamperometry (CA, “stability”).

1.2.2 Pretreatment of electrodes for the three-electrode cell

The working electrode is a 5 mm Ø glassy carbon (GC) disk electrode in PEEK sheath. Proper electrode pretreatment to get a mirror-like surface is done before every measurement. Before the first use, electrodes are sandpapered with decreasing roughness. Electrodes are polished with polishing paste (Al_2O_3 slurry; 1.0 µm and 0.05 µm) on a wet polishing cloth for 3-5 minutes, and rinsed thoroughly with water. No scratches are visible on the glassy carbon surface. Before drop-coating of catalyst, electrodes are ultrasonicated in absolute ethanol for 5 minutes, rinsed thoroughly with water, ultrasonicated in millipore water for 5 minutes, rinsed thoroughly with water and dried in an oven at 60 °C.

1.2.3 Ink preparation

Ink consists of 4 mL IPA, 960 µL H_2O and 40 µL 5% Nafion solution (binder) and 5 mg sample. The catalyst is dispersed in the solvent by 15 min of ultrasonication. Proper treatment yields a well dispersion without obvious particles. The catalyst precipitates from the dispersion over time. If any black precipitate can be seen, ultrasonication is repeated. The ink is used as soon as possible after been taken out from the ultrasonic bath.

1.2.4 Ink Dropcoating

Electrodes with 50 µg/cm² loading were produced. Consecutively the volume of 5 µL are pipetted twice onto the pretreated GC electrodes at room temperature and dried for 0.5 hours at 60 °C with a light uniform film formed on the electrode surface.

1.2.5 Conditioning

This part of the procedure consists of the determination of the open circuit potential (OCP) for 60 s and a subsequent linear sweep from the OCP to 1 V_{RHE} (5 mV/s) to avoid a harsh potential jump and stress on the working electrode. Conditioning is done by 30 cycles at 100 mV/s from 0 V_{RHE} to 1 V_{RHE}.

1.2.6 Impedance spectroscopy (determination of iR-drop)

The uncompensated resistance (R_u, iR-drop) of the system is determined by electrochemical impedance spectroscopy (EIS) at the OCP. The first step is the determination of the OCP (for 60 s). EIS is carried out at the OCP: 25 data points between 100 kHz and 10 Hz with an amplitude of 10 mV_{RHE}.

1.2.7 Cyclic voltammetry ("activity measurement")

The cell remains switched on after EIS and the potential is swept from the OCP to 1 V_{RHE} (5 mV/s). Before start of the CV, the electrode is set to rotate at 1600 rpm. Cyclic voltammetry is conducted from 1 V_{RHE} to 1.8 V_{RHE} at 5 mV/s with automatic iR-compensation using R_u from impedance spectroscopy.

1.2.8 Charge collection

The charge collection process is collected by the three-electrodes system for pellet samples which are held and dipped into the electrolyte. The potential is chosen as 2.0 V_{RHE} and the charge is directly read from the software.

1.2.9 Two-electrode cell

The two-electrode system (Hofman Cell) was controlled by using a potentiostat. An around 1.0 cm^2 Pt foil as a counter electrode was used. Electrochemical measurements were performed at 2.4 V in 0.1 M KOH. The 2.4 V include the standard reversible potential of water splitting, the resistance of the connection, the resistance of the electrolyte and the overpotentials of the both electrodes, since no reference electrode could be applied like in the three-electrode cell. The potential on the anode is therefore much smaller, similar to the cells used in industry.

1.2.10 Thermal analysis

TG-MS (thermogravimetric – mass spectrometer) analyses were performed with a NETZSCH STA 449 F3 Jupiter thermobalance setup connected to a quadrupole mass spectrometer (NETZSCH QMS 403 C Aeolos). The sample was heated under argon atmosphere with a constant gas flow of 50 mL/min from 40 to 1000°C. For measurements, a temperature program with several isothermal steps was developed to separate the thermal decomposition events of specific functional groups. A heating rate of 20 K/min was chosen to enable good signal separation and to avoid any effects of over-heating. Details are reported in literature.[2]

Methods references

1 Reiche, S., Kowalew, N. & Schlögl, R. Influence of Synthesis pH and Oxidative Strength of the Catalyzing Acid on the Morphology and Chemical Structure of Hydrothermal Carbon. *ChemPhysChem* **16**, 579-587, doi:10.1002/cphc.201402834 (2015).

2 Düngen, P., Schlögl, R. & Heumann, S. Non-linear thermogravimetric mass spectrometry of carbon materials providing direct speciation separation of oxygen functional groups. *Carbon* **130**, 614-622, doi:10.1016/j.carbon.2018.01.047 (2018).

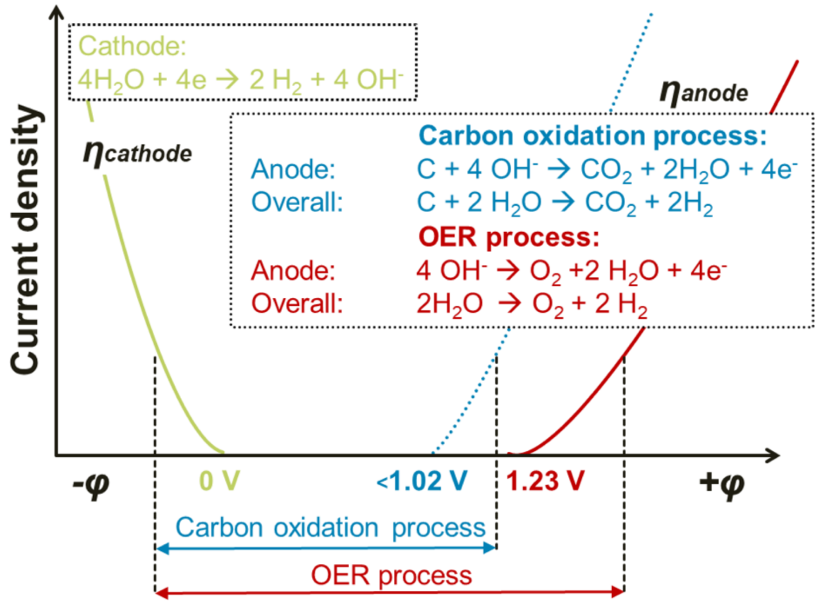

Figure S1. Schematic electrochemical polarization curves of the water splitting process using carbon as sacrificial electrode.

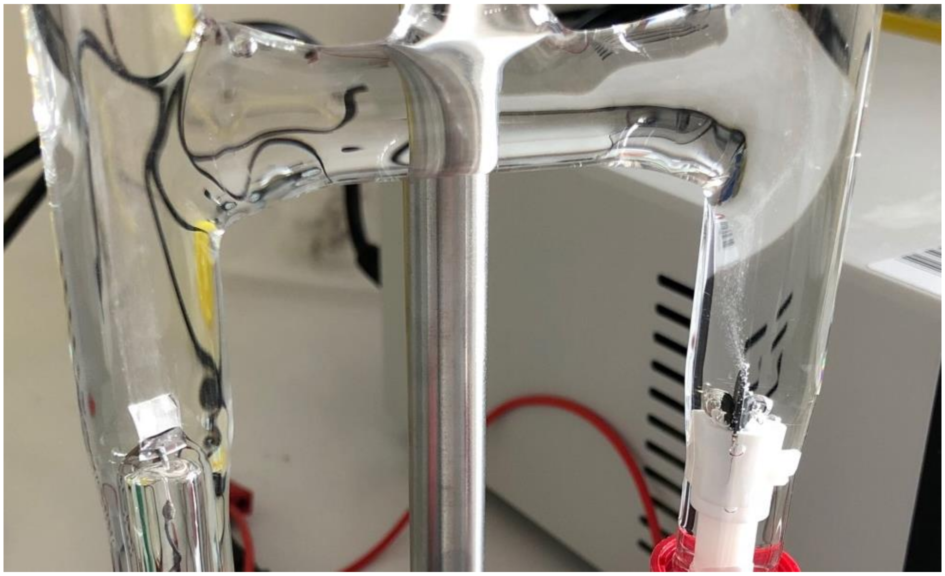

Figure S2. Hofmann system with Pt foil (left) as cathode and carbon pellet (right) as anode. The potential between the two electrodes is fixed at 2.4 V. The gas is directly collected in the two columns. At the cathode side the produced hydrogen is collected while at the anode side either oxygen from catalytic OER or CO_2 from carbon corrosion is formed. In alkaline media the produced CO_2 dissolves as carbonate.

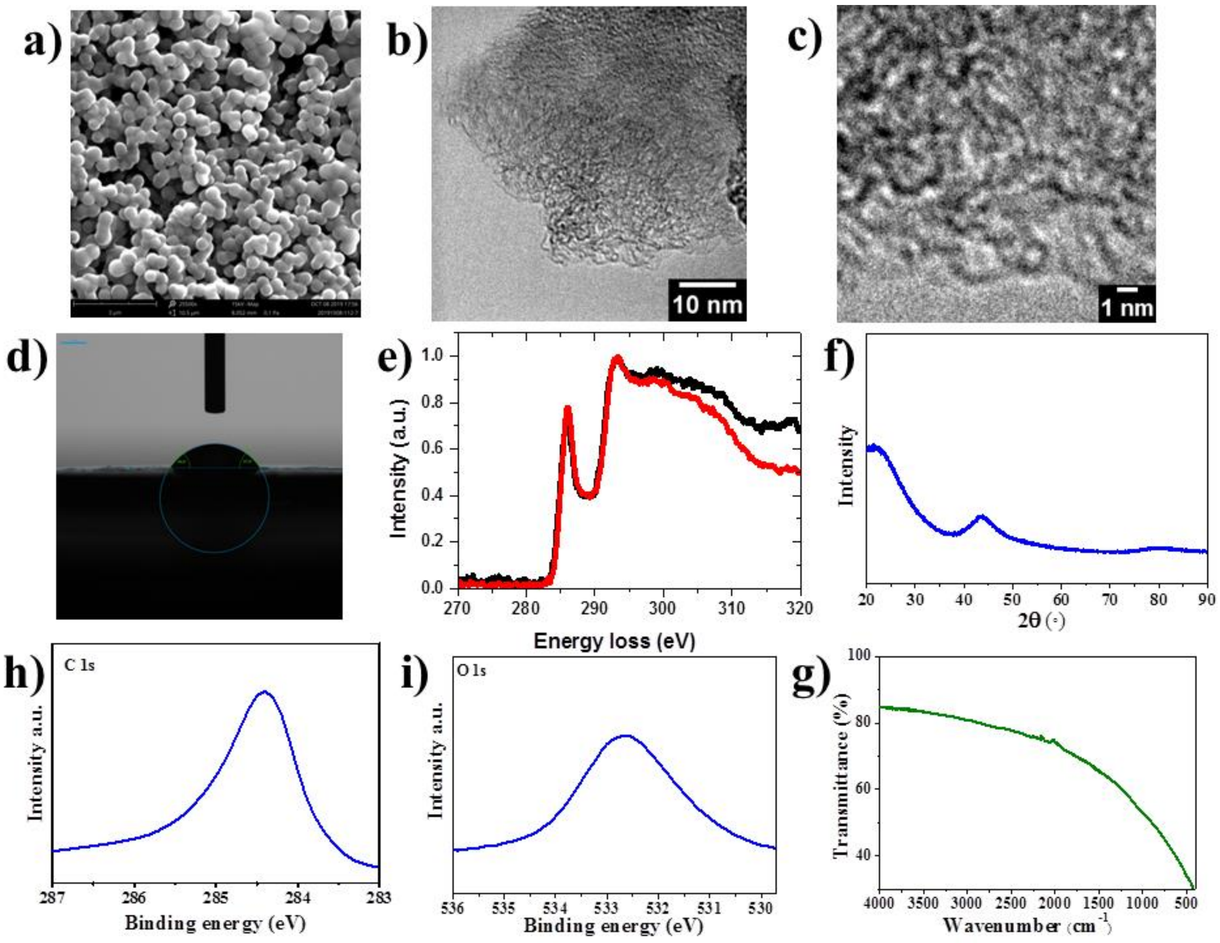

Figure S3. The characterization of the carbon pellet. a) SEM image, b) TEM image, c) high resolution TEM image, d) water angle test with an angle of 56°, e) EELS spectra with two different spots, f) XRD curves, h) XPS of C1s spectrum, i) XPS of O1s spectrum, and g) the ATR spectrum.

The pellet contains 87.4 wt% of carbon, 1.6 wt% of hydrogen and 11 wt% oxygen through the elemental analysis. The annealed carbon exhibits regular ball structure, which connected to each other. The TEM images show that the carbon is amorphous and no graphitic lattice is showing in the case, which can be confirmed by the XRD curve. The amorphous structure is favorable for the carbon surface contacting with water, showing a water contact angle of 56°. The hydrophilicity of the surface is helpful for the mass transportation during the electrolytic process.

The EELS spectra confirm the carbon is almost 100% sp2 hybridization and this indicates the graphitization process is not necessary for the formation of the sp2 hybridization. The high sp2 hybridization endows the carbon a good conductivity. The resistivity of the carbon is $45.4*10^{-5}$ $\Omega\cdot m$, which is sufficient for conducting the electrochemical reaction to some content. The functionality of the carbon surface is mainly oxygen functional groups, yet the carbon is too black to obtain IR signal. From XPS, the oxygen species on the surface can be confirmed as mainly phenol groups.

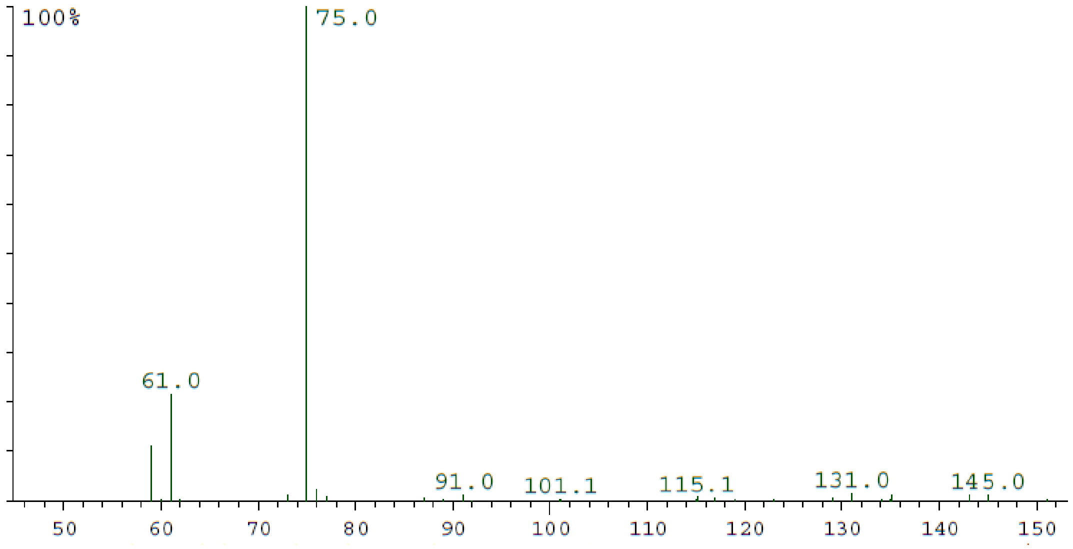

Figure S4. The mass spectrum of the electrolyte after 10-days is obtained with CH_3OH as solvent. The signal at the m/z ratio of 59 originates from the solvent. The carbonate can be detected as HCO_3^- (m/z 61) and CH_3-OCO_2^- (m/z 75). The latter is the product of the esterification of carbonate and CH_3OH, which also shows a possibility to capture the produced CO_2. The calculated CO_2 quantity from the gas analysis corresponds to 1.33 mmol. The measured decrease of OH^- in the electrolyte was determined with 0.079 mol/L to 0.063 mol/L, this corresponds to 1.66 mmol of formed carbonate. The two determined CO_2 quantities match very well. The lower excess consumption of OH^- in the electrolyte can be caused by the physical adsorption of OH^- on the carbon surface.

Potassium carbonate may precipitate as reaction progresses if the electrolyte is not exchanged. Using KOH to produce K_2CO_3 is a very beneficial industrial process. The produced CO_2 can therefore be processed in a further cycle. The saturated carbonate might also get a chance to be transferred into valuable chemicals (like CO_2 reduction) by simply change the applied potential. This will be studied in future.

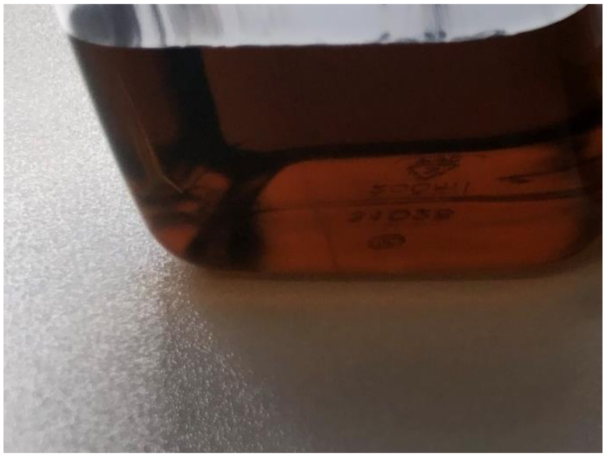

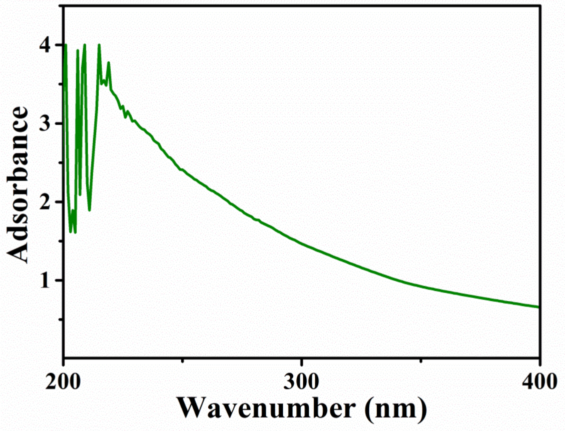

Figure S5. (left) The brownish product of the electrolyte after the pellet reacting in the two-electrode cell over 10 days. The confirmed product of the carbon anode in the electrolyte is colorless carbonate. The brownish color of the electrolyte might come from detached carbon particles from the carbon pellet. The mass spectra, does not show big amounts of other large molecular structures. Supported by the UV/Vis spectra (Ultraviolet-visible spectroscopy on a Cary Model 5000 spectrometer-Agilent) of the electrolyte (right). The broad peak corresponds to the adsorption of UV light by nanosized carbons. The structure of the detached carbon particles is very complex and also a mixture of different pieces.

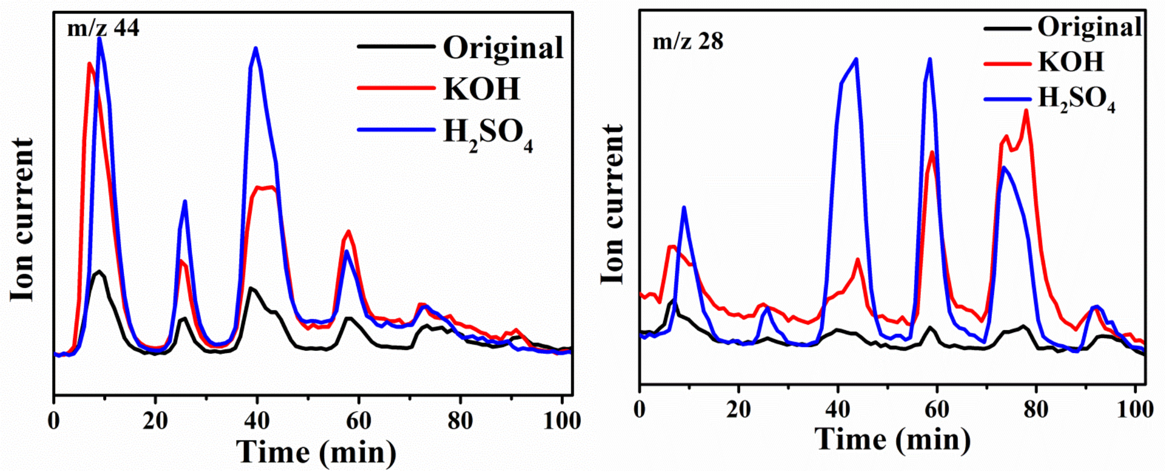

Figure S6. Thermal analysis of the original pellet, the pellet after reaction in KOH and the pellet after reaction in H_2SO_4. Ion currents of CO_2^+ (m/z 44) (left) and of CO^+ (m/z 28) (right) were determined with programmed heating rates. Based on our former work,[2] oxygen functional groups can be detected at different temperatures one by one: carboxylic group (252 °C, 10 min), lactone group on zig-zag edge (310 °C, 25 min), anhydride group (515 °C, 42 min), lactone group on armchair edge (610 °C, 58 min), phenol group (800 °C, 75min) and carbonyl group (1000 °C, 93 min). With analysis of the gas signals, a brief conclusion can be obtained. In comparison, on the carbon from acidic media, anhydride, lactone and ether groups are the dominated groups; while on the alkaline treated carbon surface, phenol groups are dominated. Phenol groups in alkaline media are easy to lose a proton forming C-O^-, which might be the combustion site. This can also explain the deep oxidation behavior because phenol groups in alkaline media are easy to be oxidized. The detail mechanism still needs to be further proved.

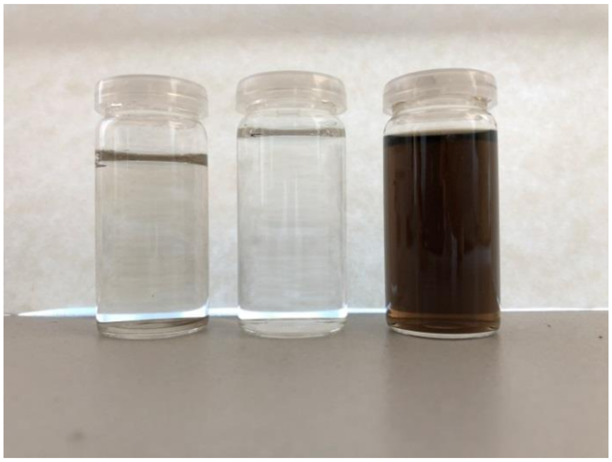

Figure S7. Different electrolytes after the electrochemical reaction: H_2SO_4 (left), K_2SO_4 (middle) and KOH (right). In acidic electrolyte, the quick functionalization passivated the carbon surface and therefore protecting the carbon pellet from physical detachment. A 30-days measurement of the pellet in acidic electrolyte was also tested, which also shows no color change of the electrolyte (Fig. S9).

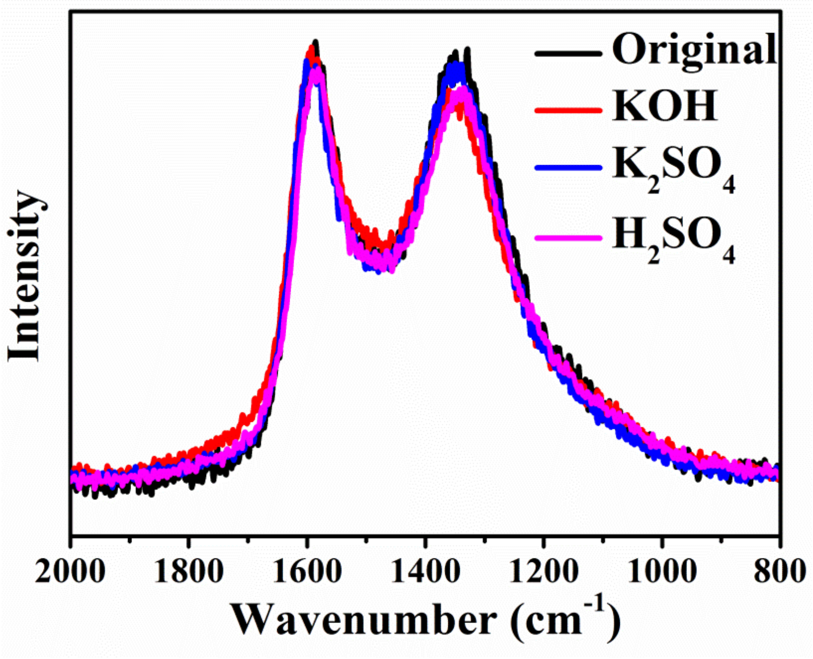

Figure S8. Raman spectra of different carbon samples. Different electrochemical behaviors lead to different functional groups on the carbon surface, when they are used in different electrolytes. However, it can be seen that after reacted in different electrolyte, the Raman peaks has no obvious change compared to the original carbon structures. This means the carbon backbones during the water splitting process has no obvious change.

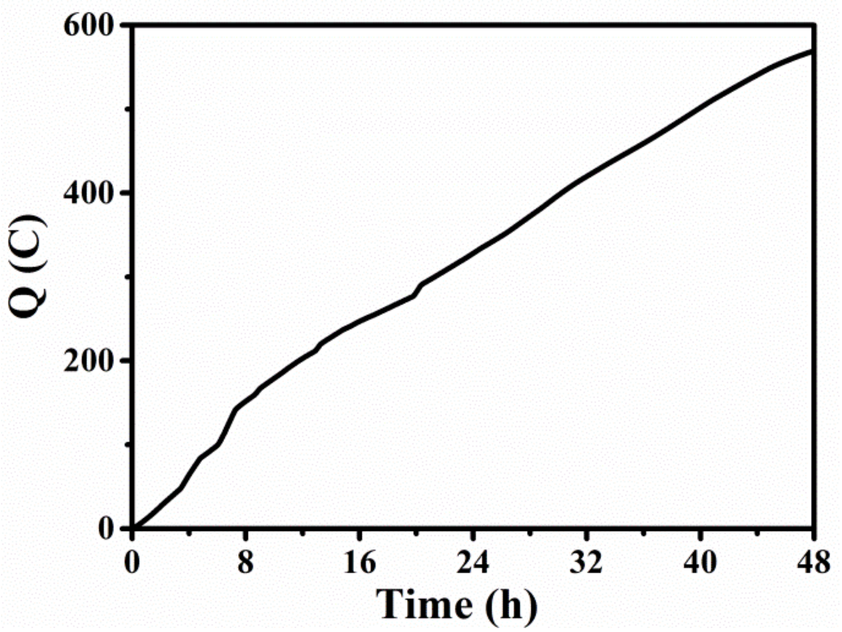

Figure S9. Charge collection of a graphite pellet in alkaline (KOH, pH 13) electrolyte. The graphite pellet has big graphene domains in layers, that begin to decompose from the edge sites.[23] When the oxidized carbon atoms are removed from the graphene edge in the form of CO_2, a fresh edge will be exposed to be continuously oxidized. Due to the perfect graphitic structure of the graphite pellet, a stable charge collection during the oxidation process at the anode can be measured. Unlike carbon derived from biomass, which usually has amorphous structures, the big pieces of the graphene are protected from physical detachment. The electrolyte after the reaction is still colorless when graphite was applied as anode material (Fig. S9), which confirms the previous explanation. As mentioned in the main text, large graphitic carbon domains can therefore reduce the detachment.

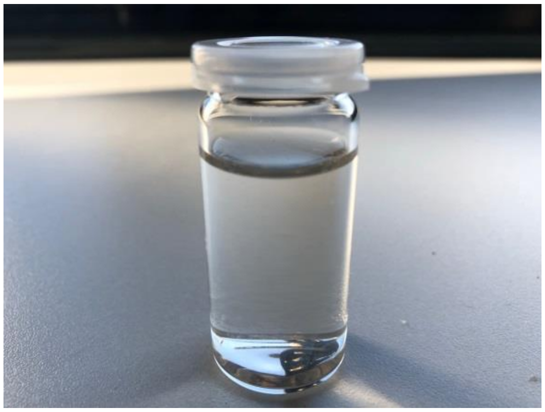

Figure S10. The KOH electrolyte after the electrocatalytic reaction of a graphite pellet.

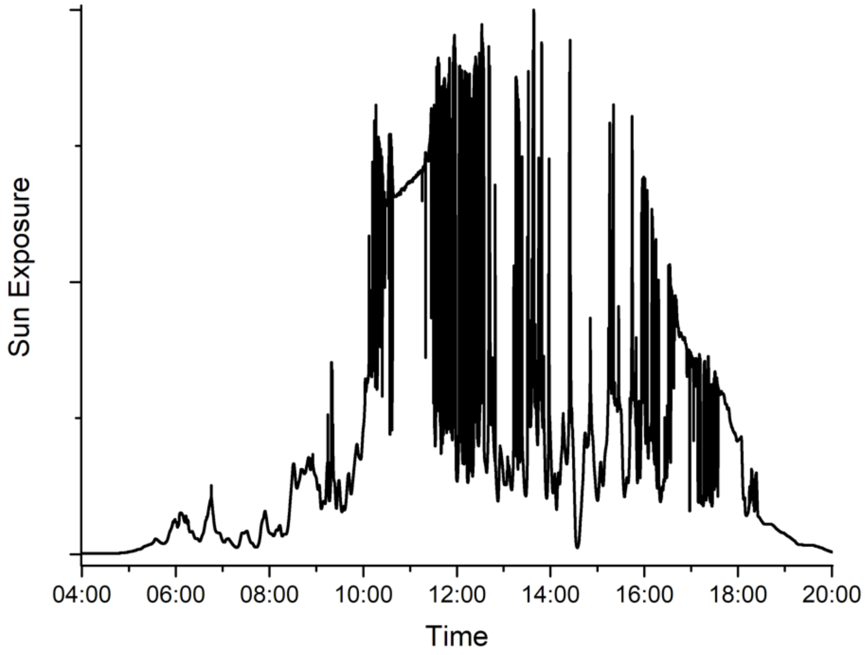

Figure S11. Fluctuating sun exposure during a cloudy day on a photovoltaic system.
